# Evaluating the Classification Accuracy of Expression Quantitative Trait Loci Calculated Polygenic Risk Scores in Alzheimer’s Disease

**DOI:** 10.3390/ijms241612799

**Published:** 2023-08-14

**Authors:** Keeley J. Brookes

**Affiliations:** Department of Biosciences, School of Science & Technology, Nottingham Trent University, Nottingham NG11 8NS, UK; keeley.brookes@ntu.ac.uk

**Keywords:** Alzheimer’s disease, polygenic risk scores, gene expression, eQTL, BDR

## Abstract

Polygenic risk scores (PRS) hold promise for the early identification of those at risk from neurodegenerative disorders such as Alzheimer’s Disease (AD), allowing for intervention to occur prior to neuronal damage. The current selection of informative single nucleotide polymorphisms (SNPs) to generate the risk scores is based on the modelling of large genome-wide association data using significance thresholds. However, the biological relevance of these SNPs is largely unknown. This study, in contrast, aims to identify SNPs with biological relevance to AD and then assess them for their ability to accurately classify cases and controls. Samples selected from the Brains for Dementia Research (BDR) were used to produce gene expression data to identify potential expression quantitative trait loci (eQTLs) relevant to AD. These SNPs were then incorporated into a PRS model to classify AD and controls in the full BDR cohort. Models derived from these eQTLs demonstrate modest classification potential with an accuracy between 61% and 67%. Although the model accuracy is not as high as some values in the literature based on significance thresholds from genome-wide association studies, these models may reflect a more biologically relevant model, which may provide novel targets for therapeutic intervention.

## 1. Introduction

Polygenic risk scores (PRS), a measure of genetic liability within an individual for a given disease, hold much promise for clinical application. These measures could be extremely useful in the early identification of individuals at risk for mid- to late-life diseases such as neurodegeneration, enabling therapeutic intervention to delay or prevent the onset of symptoms. However, given that the diseases these scores aim to predict are often complex and highly heterogeneous in their genetic (and lifestyle) aetiology, the PRS also holds the potential to identify the individual molecular pathways that are aberrant in an individual, allowing the application of precision medicine.

Alzheimer’s disease (AD), responsible for the majority of dementia cases [1], is one such disease that could benefit from the development of a robust PRS model. The onset of AD generally occurs later in life, over the age of 65, except for a small percentage that exhibits symptoms much earlier due to inherited mutations in the *APP*, *PSEN1*, and *PSEN2* genes (familial AD). The etiology of AD is complex and is a result of accumulative risk factors, both genetic and lifestyle-based. Heritability of AD is estimated to be as high as 79% [2,3], indicating a large role for genetics in the pathology; however, it is also estimated that up to a third of dementia cases may be prevented with changes in lifestyle, such as improvements in diet and activity [4]. Current treatments for AD are thought to slow the progression of the disease, with the best effects seen when drugs are administered early in diagnosis [5]. Therefore, a genetic test to run PRS analysis early in life could identify people at high genetic risk for AD, allowing intervention prior to symptom onset, resulting in increased beneficial efficacy of these drugs.

Currently, PRS are developed using data from large genome-wide association studies (GWAS). Single Nucleotide Polymorphisms (SNP) models are derived from selecting SNPs associated with AD under a set significance threshold and calculating risk scores based on the occurrence of effect alleles and their effect sizes to increase or decrease the risk for AD. These models are then tested for their accuracy in classifying individuals with AD from controls [6]. The SNPs included in these models range from a few hundred to thousands and provide varying levels of accuracy [7]; however, what these SNPs have in common is that the vast majority are non-coding, with no discernible biological consequence. The development of PRS is still in its infancy, with multiple limitations being highlighted. SNPs are selected based on statistical association studies rather than knowledge of biological consequences. The estimated effect sizes of some SNPs included in these models are often negligible and therefore debatable as to whether they play a significant and therapeutically relevant role in the disease’s aetiology. Furthermore, it is thought that some SNPs with important biological effects may be omitted from the models because their statistical significance does not reach the threshold [8,9,10].

An alternative approach utilising SNPs with known biological effects, such as influencing gene expression, may provide a more clinically relevant PRS model. This clinically relevant model will aid in the understanding of biological consequences ranging from genetic variation to pathological change. This knowledge could be crucial for identifying new avenues for therapeutic intervention given specific biological pathway aberrations. In addition, it may provide a more accurate and replicable model for clinical application, identifying people at high risk of developing AD before symptom onset, allowing early therapeutic intervention, and preventing the disease altogether.

This study attempts to advance current PRS analysis in AD by utilising gene expression data to inform SNP selection for the model, in contrast to the significance of association for SNPs in GWAS. Expression Quantitative Trait Loci (eQTL) were identified using gene expression data derived from RNA-sequencing in post-mortem brains and genotype data of AD (*n* = 8) and control (*n* = 8) individuals from the Brains for Dementia Research (BDR) cohort. These eQTL SNPs were used to create a PRS model to classify AD and controls in the full BDR cohort [11].

## 2. Results

RNA-sequencing data was used to determine gene expression levels in 16 post-mortem frontal cortex samples (8 = AD; 8 = control) taken from the BDR cohort. Gene expression counts suggested the expression of 22,117 genes in the bulk frontal cortex tissue, with 1844 genes demonstrating significant gene expression differences between the AD and control samples.

Additive linear model eQTL analysis with all SNPs (*n* = 283,464) available for the BDR cohort [11] and all genes expressed (*n* = 22,117) yielded 766,358 SNP-gene pairs with a False Discovery Rate (FDR) adjusted *p* value < 0.01. These SNP-gene pairs consisted of 41,378 unique SNPs as potential eQTLs to utilise in the PRS model. SNP IDs were matched with the GWAS summary statistics of three commonly used datasets: Lambert (International Genomics of Alzheimer’s Project (IGAP)) [12], Jansen [13], and Bellenguez [14]. The commonality of SNPs between these datasets and the BDR varied (Table 1), with the Jansen dataset having the highest commonality of SNPs with the BDR dataset. Initial analyses of these SNP models yielded moderate classification accuracy with Area Under the Curve (AUC) statistics between 61–64%. The samples used to identify eQTLs were all *APOE* ε3 homozygotes, controlling for the large effect size exhibited by the two *APOE* isoform SNPs (rs429358 and rs7412). Effect sizes for these two SNPs were only available in the IGAP and Jansen summary statistics and were added to the eQTL models, leading to an increase in significance and classification accuracy of up to 67% (Table 1).

The eQTL SNP-gene pairs were limited to only those genes (442 out of 1844) that displayed significant (adjusted *p* value < 0.05) differential expression (DE) between the AD and control samples, ensuring disease relevant SNPs were included in the model and reducing the number of SNP-gene pairs to 21,709 with 3650 unique SNPs for the eQTL-DE PRS models (Table 1). PRS generated with these SNPs did not show a significant correlation with AD and had poor classification accuracies of 53–55%. However, the addition of the *APOE* isoform SNPs to the models increased classification accuracy to ~65% (Table 1).

**Figure 1:**
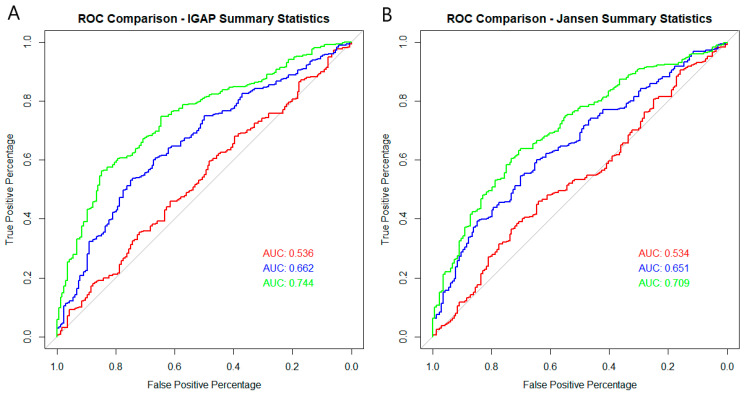
Receiver Operating Characteristic (ROC) curves for PRS were generated for the BDR cohort using the IGAP (**A**) and Jansen (**B**) summary statistics. Utilising eQTL SNPs that governed genes found to be differentially expressed in the RNA-sequencing dataset produced a small and non-significant classification curve (red). The classification improved with the addition of the two *APOE* isoform SNPs (blue) but did not reach the accuracy of classification achieved when using the thresholding model generated with SNPs with significance equal to or less than 5 × 10^−8^ in the base dataset, excluding 500 kb around the *APOE* locus but including the *APOE* isoform SNPs (green).

For comparison purposes, PRS models containing just the *APOE* isoform SNPs and the best model obtained from the “thresholding” approach were conducted. A PRS calculated using the two *APOE* isoform SNPs produced a highly significant and consistent classification accuracy of 70% regardless of which dataset was used to derive the effect sizes from. Thresholding models excluding the *APOE* region (500 kb surrounding the locus, hg19: 45,160,844–45,660,844) provided classification estimates of around 60%, which increased to >70% with the addition of the two *APOE* isoform SNPs. Interestingly, the best classification models were those that included all SNPs with a *p* value ≤ 5 × 10^−8^, without the removal of 500 kb surrounding the *APOE* locus (Table 1, Figure 1).

Exploration of the SNP-gene pairs identified from the eQTL-DE analyses (Supplemental Tables S1 and S2) found several SNPs that were located within several known risk factor genes for AD, including *CR1*, *PICALM*, *MEF2C*, *CASS4*, *SLC24A4/RIN3*, *INPP5D* [12], *CLNK*, *CNTNAP2*, *ABI3*, *PLCG2* [13,15], *BLNK*, *JAZF1*, *ABCA1*, *TMEM106B* [14]. However, the gene expression affected by these SNPs was often on a different chromosome, with only 14 of the DE SNP-gene pairs occurring on the same chromosome and within 1 Mb of the transcription start/stop sites of the named gene (Supplemental Table S2). Interestingly, several DE genes were consistently regulated by the same set of SNPs consisting of these known associated AD candidate genes and displayed higher expression in the AD samples compared to the controls (Supplemental Tables S2 and S5). These genes include three tumour suppressors (*DMBT1*, *MTUS1*, and *KLRC1*), two genes involved with cell communication and myelin (*GJB1* and *KLK6*), and a P450 monooxygenase involved in the synthesis of cholesterol and lipids (*CYP4F12*).

## 3. Discussion

This study aimed to explore a different strategy for determining SNPs to be included in PRS models to classify AD samples from controls. Utilising the BDR cohort genotype and gene expression data, eQTL SNPs were identified for genes that displayed significant differential expression between AD and control post-mortem frontal cortex tissue. All samples were *APOE* isoform ε3 homozygous to identify key AD pathology regulators outside of this main risk factor gene. Out of the 1844 genes observed to be differentially expressed, 442 genes were shown to be correlated with genotypes, indicating that some gene expression differences observed may be due to other factors (lifestyle) or consequences of disease state. These eQTLs were then used to calculate a PRS using the PLINK scoring algorithm, with the PRS being subjected to a logistic regression to determine if a higher score correlated to a higher probability of a sample being classified as a case. The accuracy of this was determined from the AUC statistics derived from ROC curves. The study indicates that moderate classification accuracy (~65%) can be obtained from eQTLs for differentially expressed genes in AD when the *APOE* isoform SNPs are included in the model.

The majority of eQTL effects were seen on different chromosomes than where the SNP resides; in fact, only 14 SNP-gene pairings from the eQTL-DE analyses were found to be on the same chromosome and within 1 Mb of the transcription start/stop site of the named gene. In contrast to suggesting that these SNPs do not act on genes near-by, it could suggest that the *cis* effects of eQTLs are subtle and that large sample sizes are required to detect them. What is being detected in this study are the further downstream (larger) effects of a biological pathway initiated at the base change. Reminiscent of a “butterfly effect”, where a single base change can lead to a cascade of changes, leading to increasingly larger effect sizes down the biological pathway that can be more readily detected [16,17].

Several genes appeared to be coordinated by the same set of SNPs, including those in well-known AD candidate risk genes; interestingly, the direction of change in expression of these genes was the same, indicating higher expression in AD brains than in controls. Three genes are known tumour suppressors, and so one could speculate that increased expression correlates with increased cell death, which may support the inverse correlation between cancer and Alzheimer’s disease observed in several studies [18]. Other genes identified in this set were *GJB1* and *KLK6,* which function in cell communication and myelin turnover. Whereas the data presented here supports that of an earlier study observing increased levels of KLK6 in the plasma of AD patients [19], the increase in expression of *GJB1* (which encodes for connexin-32) in the AD group contradicts other studies that suggest a decrease in connexins in AD mouse models [20]. Finally, *CYP4F12* is a member of the p450 cytochrome family, involved in metabolism and cholesterol and lipid pathways, which has also been observed to be upregulated in AD [21], with further evidence for their role in neurodegeneration supported by other P450 pathway components also showing to be elevated in AD [22,23]. It is worth noting that differences in gene expression between a disease state and a control cannot infer causality, as some gene expression changes may be consequences of the disease state rather than causing it. However, the connection presented here between known AD risk gene polymorphisms and changes in gene expression warrants further investigation to shed light on the biological pathways between genetic risk variants and pathology.

PRS models based on eQTLs do not have better overall accuracy than those using the *APOE* isoform SNPs alone in the model or in conjunction with models obtained using a sequential threshold approach. The best PRS models presented in this study came from the “Thresholding” approach, utilising all SNPs, including those in the *APOE* locus. Interestingly, the best threshold models achieved using the IGAP and Jansen datasets utilised SNPs with a *p* value of <5 × 10^−8^; whereas the best model achieved using the Bellenguez dataset used a much higher significance threshold of *p* ≤ 0.2917 and therefore a greater number of SNPs. This highlights that the choice of summary statistics to create PRS may influence the outcome of the model given the differing overlaps of SNPs, effect sizes, and significance values across datasets. In contrast, it is interesting to note that the effect sizes of the *APOE* isoform SNPs differ greatly between the summary statistics from the IGAP [12] and Jansen [13] datasets (rs429358: 1.35 vs. 0.162; rs7412: −0.387 vs. −0.885, respectively—Supplemental Tables S3 and S4). However, the accuracy of the classification is very similar, indicating that the allele frequency difference in the target dataset is the main influence on the classification accuracy, which has also been shown in a previous study [24].

In comparison to other PRS studies in the literature that utilise AUC statistics, the classification accuracy of the *APOE* isoform SNPs alone seems consistent, with AUCs ranging from 68–70% [25,26,27,28]. This study is also consistent in its finding that PRS generated from SNPs from outside the *APOE* region demonstrate AUCs in the range of ~55–60% with SNP numbers ranging from the GWAS “hits” to several thousand [25,26,28,29]. In contrast, other studies have achieved much higher classification accuracies, reaching over 80% [30,31]. In both studies, thresholding was used to select SNPs for the PRS from the IGAP summary statistic dataset, including those with a liberal significance value of *p* ≤ 0.5, leading to SNP numbers akin to those in all eQTL analyses presented here. These studies demonstrate that highly predictive models are possible when including SNPs with small but important effects, though whether these will be actionable targets for therapeutic drugs or lead to clear precision medical intervention is hard to say, and therefore a trade-off between predictability and clinical practicality may be warranted.

The PRS model incorporating eQTL SNPs for genes that were identified as significantly differently expressed displayed a range of classification accuracies from 53–55%, which failed to make significance cut-offs. This is likely due to the sample size, as the expression work utilised only 16 samples and therefore lacked the power to detect more subtle gene expression differences. It is likely that with a larger sample, more differentially expressed genes would be observed, increasing the number of eQTLs in the model and the classification accuracy. This is supported by the observation that using all eQTLs produced greater classification accuracy than those in DE genes alone and may have included disease specific eQTLs that govern gene expression differences that did not meet the DE significance cut-off. It is also possible that not all AD-related SNPs act in the brain, and therefore key predictive SNPs would be missing from this analysis, leading to the lower classification accuracy observed.

Although the sample size here is too small to calculate meaningful effect sizes of changes in transcription level, a risk score based on these may represent a more accurate, biologically relevant, and translatable across ancestry classification method [32,33,34]. Recent studies suggest that classification based on Transcription Risk Scores (TRS) outperforms those based on genetic risk scores [32,34]. Though the exact method as to how to calculate the scores differs, it is thought that developing a multi-SNP-based predictor of gene expression may help link GWAS findings to functional outcomes and better prediction for disease that may be more translatable across different datasets and ancestry groups than individual SNP-based tests [33].

## 4. Materials and Methods

The BDR cohort is a longitudinal clinicopathological project [35] that has complimentary whole genome genotype information for genetic analysis obtained from the Illumina Neurochip aligned to hg19 SNP coordinates [11].

Sixteen samples were selected from the BDR cohort (Oxford Brain Bank’s generic REC approval 15/SC/0639) for RNA-sequencing. All samples were neuropathologically confirmed AD cases (*n* = 8) or cognitively normal controls (*n* = 8) with no other neuropathology. Samples were matched on biological sex, age at death (*p* = 0.69), and PMI (*p* = 0.67), and all samples were homozygous for the *APOE* isoform ε3. RNA was extracted from bulk frontal-cortex tissue using previously established protocols [36]. In total, 20 ng of the total RNA per sample were provided to the UCL Genomics Facility (London, UK) for Kapa mRNA HyperPrep library preparation and sequenced on the Illumina NextSeq 2000, generating ~30 M paired end reads per sample for analysis.

Raw reads provided by the UCL Genomics Facility were assessed for quality using FASTQC and aligned to the hg19 human reference genome using HISAT2 [37]. Binary alignment files were filtered for mapped pair-reads with a phred score ≥ 30, before generating read counts per gene with the ‘Featurecounts’ programme [38]. Differential gene expression was identified using DESeq2, filtering for low gene counts [39].

Gene count data generated was used alongside genotype information to identify eQTLs using the R package ‘Matrix eQTL’ [40]. Briefly, gene count, gene location, and genotype data in the form of minor allele counts and corresponding SNP loci information were used to model the effect of genotype in an additive linear model, with the FDR significance threshold set at 0.01. Both cis and trans eQTLs were considered.

SNPs identified as eQTLs were used to generate PRS on the full BDR cohort of AD (*n* = 356) and control (*n* = 164) samples using the –score command in PLINK v1.9 [41]. Multiple summary statistic datasets were obtained to provide effect sizes for the PRS calculations; this was to ensure that the maximum number of SNPs from the eQTL analysis could be captured in the PRS. Summary statistics produced from three large GWAS were utilized—IGAP, Jansen, and Bellenguez [12,13,14]. Each dataset was clumped using the 1000Genomes European dataset in PLINK v1.9 [41], using the parameters –clump-p1 1; –clump-p2 1; –clump-kb 250; and –clump-r2 0.8. The eQTL SNPs were matched with these SNPs, and where multiple SNPs were found to reside in the same clump, the most significant SNP was taken forward into the PRS.

Thresholding models were generated by including SNPs present in both the summary statistics from the three GWAS datasets and the BDR dataset. SNPs were incorporated into the model at increasing significance levels based on the *p* value for association in the GWAS dataset. The threshold for inclusion started at *p* ≤ 5 × 10^−8^ and increased at intervals of 10^−6^ (millionths) until all the SNPs were incorporated (*p* = 1).

Scores generated in PLINK were assessed in R [42] using logistic regression and the ‘pROC’ package [43]. The generation of area under the curve (AUC) statistics arising from the receiver operating characteristic (ROC) curves provided estimates of the models’ overall accuracy of case-control classification.

## 5. Conclusions

In conclusion, this study and emerging investigations from the literature would indicate that utilising SNPs that have functional effects may be a more robust method to identify key DNA variants that could predict disease across populations. Further to this, elucidation of these variants would also uncover disease aetiology, identify key targets for intervention, and lead to precision medicine guided by genetics.

## Figures and Tables

**Table 1:** Summary results from PRS models developed from SNPs found to be eQTLs for gene expression in human post-mortem frontal brain tissue. Initial risk scores were generated from those SNPs that were found to be eQTLs in the entire dataset (*n* = 41,378 SNPs); these were then restricted to only those that influenced genes found to be differentially expressed between controls and AD samples (*n* = 3650 SNPs). Samples used to generate gene expression data were *APOE* ε3 homozygous and therefore excluded the effect of the isoform SNPs (rs429358 and rs7412); these were added into the model to generate scores on the entire BDR cohort. The classification of AD and controls was found to be ~65–67% accurate using these models. Comparison with just the *APOE* SNPs and the best thresholding models yielded more significant results.

		eQTLs	eQTLs Plus APOE	eQTLs in DE Genes	eQTLs in DE Genes Plus APOE	rs429358 & rs7412 Only	Best Model Using Thresholding without APOE Region	Best Model Using Thresholding Plus APOE Isoform SNPs	Best Model Using Thresholding with APOE Region
**IGAP**	**# SNPs**	17,865	17,867	1614	1616	2	29	31	56
	**Logisitic Regression *p* value**	9.22 × 10^−6^	1.52 × 10^−8^	0.184	3.18 × 10^−8^	6.89 × 10^−15^	6.73 × 10^−5^	2.61 × 10^−16^	9.20 × 10^−18^
	**Area Under the Curve**	0.6144	0.6508	0.5357	0.6616	0.7082	0.6078	0.7442	0.7633
**Jansen**	**# SNPs**	34,894	34,896	3116	3118	2	62	64	164
	**Logisitic Regression *p* value**	1.02 × 10^−6^	2.51 × 10^−9^	0.264	7.72 × 10^−8^	2.1 × 10^−14^	0.0002	1.93 × 10^−12^	3.72 × 10^−16^
	**Area Under the Curve**	0.6417	0.6738	0.5335	0.6511	0.7083	0.6033	0.7089	0.7543
**Bellenguez**	**# SNPs**	30,863	-	2759	-	-	70,674	-	-
	**Logisitic Regression *p* value**	1.76 × 10^−5^	-	0.03	-	-	2.73 × 10^−11^	-	-
	**Area Under the Curve**	0.6241	-	0.5586	-	-	0.6865	-	-

## Data Availability

Genetic data and raw RNA-sequencing reads are available via the Dementias Platform UK Data Portal (https://portal.dementiasplatform.uk/).

## Supplementary Materials

The following supporting information can be downloaded at: https://www.mdpi.com/article/10.3390/ijms241612799/s1.

## References

[1] Barker W.W., Luis C.A., Kashuba A., Luis M., Harwood D.G., Loewenstein D., Waters C., Jimison P., Shepherd E., Sevush S. (2002). Relative Frequencies of Alzheimer Disease, Lewy Body, Vascular and Frontotemporal Dementia, and Hippocampal Sclerosis in the State of Florida Brain Bank. Alzheimer Dis. Assoc. Disord..

[2] Gatz M., Reynolds C.A., Fratiglioni L., Johansson B., Mortimer J.A., Berg S., Fiske A., Pedersen N.L. (2006). Role of Genes and Environments for Explaining Alzheimer Disease. Arch. Gen. Psychiatry.

[3] Sims R., Hill M., Williams J. (2020). The Multiplex Model of the Genetics of Alzheimer’s Disease. Nat. Neurosci..

[4] Livingston G., Huntley J., Sommerlad A., Ames D., Ballard C., Banerjee S., Brayne C., Burns A., Cohen-Mansfield J., Cooper C. (2020). Dementia Prevention, Intervention, and Care: 2020 Report of the Lancet Commission. Lancet.

[5] Sims J.R., Zimmer J.A., Evans C.D., Lu M., Ardayfio P., Sparks J., Wessels A.M., Shcherbinin S., Wang H., Nery E.S.M. (2023). Donanemab in Early Symptomatic Alzheimer Disease: The TRAILBLAZER-ALZ 2 Randomized Clinical Trial. JAMA.

[6] Harrison J.R.J.R., Mistry S., Muskett N., Escott-Price V., Brookes K. (2020). From Polygenic Scores to Precision Medicine in Alzheimer’s Disease: A Systematic Review. J. Alzheimer’s Dis..

[7] Rowe T.W., Katzourou I.K., Stevenson-Hoare J.O., Bracher-Smith M.R., Ivanov D.K., Escott-Price V. (2021). Machine Learning for the Life-Time Risk Prediction of Alzheimer’s Disease: A Systematic Review. Brain Commun..

[8] Janssens A.C.J.W. (2019). Validity of Polygenic Risk Scores: Are We Measuring What We Think We Are?. Hum. Mol. Genet..

[9] Cecile A., Janssens J.W., Joyner M.J. (2019). Polygenic Risk Scores That Predict Common Diseases Using Millions of Single Nucleotide Polymorphisms: Is More, Better?. Clin. Chem..

[10] Martens F.K., Tonk E.C.M., Janssens A.C.J.W. (2019). Evaluation of Polygenic Risk Models Using Multiple Performance Measures: A Critical Assessment of Discordant Results. Genet. Med..

[11] Young J., Gallagher E., Koska K., Guetta-Baranes T., Morgan K., Thomas A., Brookes K.J. (2021). Genome-Wide Association Findings from the Brains for Dementia Research Cohort. Neurobiol. Aging.

[12] Lambert J.-C., Ibrahim-Verbaas C.A., Harold D., Naj A.C., Sims R., Bellenguez C., Jun G., DeStefano A.L., Bis J.C., Beecham G.W. (2013). Meta-Analysis of 74,046 Individuals Identifies 11 New Susceptibility Loci for Alzheimer’s Disease. Nat. Genet..

[13] Jansen I.E., Savage J.E., Watanabe K., Bryois J., Williams D.M., Steinberg S., Sealock J., Karlsson I.K., Hägg S., Athanasiu L. (2019). Genome-Wide Meta-Analysis Identifies New Loci and Functional Pathways Influencing Alzheimer’s Disease Risk. Nat. Genet..

[14] Bellenguez C., Küçükali F., Jansen I.E., Kleineidam L., Moreno-Grau S., Amin N., Naj A.C., Campos-Martin R., Grenier-Boley B., Andrade V. (2022). New Insights into the Genetic Etiology of Alzheimer’s Disease and Related Dementias. Nat. Genet..

[15] Sims R., Van Der Lee S.J.J., Naj A.C.C., Bellenguez C., Badarinarayan N., Jakobsdottir J., Kunkle B.W.W., Boland A., Raybould R., Bis J.C.C. (2017). Rare Coding Variants in PLCG2, ABI3, and TREM2 Implicate Microglial-Mediated Innate Immunity in Alzheimer’s Disease. Nat. Genet..

[16] Hart J.R., Zhang Y., Liao L., Ueno L., Du L., Jonkers M., Yates J.R., Vogt P.K. (2015). The Butterfly Effect in Cancer: A Single Base Mutation Can Remodel the Cell. Proc. Natl. Acad. Sci. USA.

[17] Desi N., Tay Y. (2019). The Butterfly Effect of RNA Alterations on Transcriptomic Equilibrium. Cells.

[18] Ospina-Romero M., Glymour M.M., Hayes-Larson E., Mayeda E.R., Graff R.E., Brenowitz W.D., Ackley S.F., Witte J.S., Kobayashi L.C. (2020). Association Between Alzheimer Disease and Cancer With Evaluation of Study Biases: A Systematic Review and Meta-Analysis. JAMA Netw. Open.

[19] Patra K., Soosaipillai A., Sando S.B., Lauridsen C., Berge G., Møller I., Grøntvedt G.R., Bråthen G., Begcevic I., Moussaud S. (2018). Assessment of Kallikrein 6 as a Cross-Sectional and Longitudinal Biomarker for Alzheimer’s Disease. Alzheimers Res. Ther..

[20] Angeli S., Kousiappa I., Stavrou M., Sargiannidou I., Georgiou E., Papacostas S.S., Kleopa K.A. (2020). Altered Expression of Glial Gap Junction Proteins Cx43, Cx30, and Cx47 in the 5XFAD Model of Alzheimer’s Disease. Front. Neurosci..

[21] Cioffi F., Hassan R., Adam I., Bansal R., Broersen K. (2021). A Review of Oxidative Stress Products and Related Genes in Early Alzheimer’s Disease. J. Alzheimer’s Dis..

[22] Borkowski K., Pedersen T.L., Seyfried N.T., Lah J.J., Levey A.I., Hales C.M., Dammer E.B., Blach C., Louie G., Kaddurah-Daouk R. (2021). Association of Plasma and CSF Cytochrome P450, Soluble Epoxide Hydrolase, and Ethanolamide Metabolism with Alzheimer’s Disease. Alzheimers Res. Ther..

[23] Sarparast M., Dattmore D., Alan J., Lee K.S.S. (2020). Cytochrome P450 Metabolism of Polyunsaturated Fatty Acids and Neurodegeneration. Nutrients.

[24] Brookes K.J., Guetta-Baranes T., Thomas A., Morgan K. (2023). An Alternative Method of SNP Inclusion to Develop a Generalized Polygenic Risk Score Analysis across Alzheimer’s Disease Cohorts. Front. Dement..

[25] Yokoyama J.S., Bonham L.W., Sears R.L., Klein E., Karydas A., Kramer J.H., Miller B.L., Coppola G. (2015). Decision Tree Analysis of Genetic Risk for Clinically Heterogeneous Alzheimer’s Disease. BMC Neurol..

[26] Sleegers K., Bettens K., De Roeck A., Van Cauwenberghe C., Cuyvers E., Verheijen J., Struyfs H., Van Dongen J., Vermeulen S., Engelborghs S. (2015). A 22-Single Nucleotide Polymorphism Alzheimer’s Disease Risk Score Correlates with Family History, Onset Age, and Cerebrospinal Fluid Abeta42. Alzheimers Dement..

[27] Escott-Price V., Sims R., Bannister C., Harold D., Vronskaya M., Majounie E., Badarinarayan N., Morgan K., Passmore P., Holmes C. (2015). Common Polygenic Variation Enhances Risk Prediction for Alzheimer’s Disease. Brain.

[28] Leonenko G., Baker E., Stevenson-Hoare J., Sierksma A., Fiers M., Williams J., de Strooper B., Escott-Price V. (2021). Identifying Individuals with High Risk of Alzheimer’s Disease Using Polygenic Risk Scores. Nat. Commun..

[29] Tosto G., Bird T.D., Tsuang D., Bennett D.A., Boeve B.F., Cruchaga C., Faber K., Foroud T.M., Farlow M., Goate A.M. (2017). Polygenic Risk Scores in Familial Alzheimer Disease. Neurology.

[30] Escott-Price V., Myers A.J., Huentelman M., Hardy J. (2017). Polygenic Risk Score Analysis of Pathologically Confirmed Alzheimer’s Disease. Ann. Neurol..

[31] Escott-Price V., Myers A., Huentelman M., Shoai M., Hardy J., Hardy J. (2019). Polygenic Risk Score Analysis of Alzheimer’s Disease in Cases without APOE4 or APOE2 Alleles. J. Prev. Alzheimer’s Dis. JPAD.

[32] Marigorta U.M., Denson L.A., Hyams J.S., Mondal K., Prince J., Walters T.D., Griffiths A., Noe J.D., Crandall W.V., Rosh J.R. (2017). Transcriptional Risk Scores Link GWAS to EQTL and Predict Complications in Crohn’s Disease HHS Public Access Author Manuscript. Nat. Genet..

[33] Liang Y., Pividori M., Manichaikul A., Palmer A.A., Cox N.J., Wheeler H.E., Im H.K. (2022). Polygenic Transcriptome Risk Scores (PTRS) Can Improve Portability of Polygenic Risk Scores across Ancestries. Genome Biol..

[34] Pain O., Glanville K.P., Hagenaars S., Selzam S., Fürtjes A., Coleman J.R.I., Rimfeld K., Breen G., Folkersen L., Lewis C.M. (2021). Imputed Gene Expression Risk Scores: A Functionally Informed Component of Polygenic Risk. Hum. Mol. Genet..

[35] Francis P.T., Costello H., Hayes G.M. (2018). Brains for Dementia Research: Evolution in a Longitudinal Brain Donation Cohort to Maximize Current and Future Value. J. Alzheimer’s Dis..

[36] Chappell S., Patel T., Guetta-Baranes T., Sang F., Francis P.T., Morgan K., Brookes K.J. (2018). Observations of Extensive Gene Expression Differences in the Cerebellum and Potential Relevance to Alzheimer’s Disease. BMC Res. Notes.

[37] Kim D., Langmead B., Salzberg S.L. (2015). HISAT: A Fast Spliced Aligner with Low Memory Requirements. Nat. Methods.

[38] Liao Y., Smyth G.K., Shi W. (2014). FeatureCounts: An Efficient General Purpose Program for Assigning Sequence Reads to Genomic Features. Bioinformatics.

[39] Love M.I., Huber W., Anders S. (2014). Moderated Estimation of Fold Change and Dispersion for RNA-Seq Data with DESeq2. Genome Biol..

[40] Shabalin A.A. (2012). Gene Expression Matrix EQTL: Ultra Fast EQTL Analysis via Large Matrix Operations. Bioinformatics.

[41] Purcell S., Neale B., Todd-Brown K., Thomas L., Ferreira M.A., Bender D., Maller J., Sklar P., de Bakker P.I., Daly M.J. (2007). PLINK: A Tool Set for Whole-Genome Association and Population-Based Linkage Analyses. Am. J. Hum. Genet..

[42] R Core Team (2021). R: A Language and Environment for Statistical Computing.

[43] Robin X., Turck N., Hainard A., Tiberti N., Lisacek F., Sanchez J.-C., Müller M. (2011). PROC: An Open-Source Package for R and S+ to Analyze and Compare ROC Curves. BMC Bioinform..

